# Development of a highly effective low-cost vaporized hydrogen peroxide-based method for disinfection of personal protective equipment for their selective reuse during pandemics

**DOI:** 10.1186/s13099-020-00367-4

**Published:** 2020-06-19

**Authors:** Vikram Saini, Kriti Sikri, Sakshi Dhingra Batra, Priya Kalra, Kamini Gautam

**Affiliations:** 1 Laboratory of Infection Biology and Translational Research, Department of Biotechnology, All India Institute of Medical Sciences (AIIMS), New Delhi, 110029 India; 2 Biosafety Laboratory-3 Centralized Core Research Facility (CCRF), All India Institute of Medical Sciences (AIIMS), New Delhi, 110029 India

**Keywords:** COVID-19, Personal protective equipment, Vaporized hydrogen peroxide, *Bacillus stearothermophilus* spores

## Abstract

**Background:**

Personal Protective Equipment (PPE) is required to safely work with biological agents of bacterial (i.e. *Mycobacterium tuberculosis)* or viral origin (Ebola and SARS). COVID-19 pandemic especially has created unforeseen public health challenges including a global shortage of PPE needed for the safety of health care workers (HCWs). Although sufficient stocks of PPE are currently available, their critical shortage may develop soon due to increase in demand and depletion of existing supply lines. To empower our HCWs and ensure their continued protection, proactive measures are urgently required to develop procedures to safely decontaminate the PPEs to allow their “selective reuse” during contingency situations.

**Methods:**

Herein, we have successfully developed a decontamination method based on vaporized hydrogen peroxide (VHP). We have used a range of concentration of hydrogen peroxide to disinfect PPE (coveralls, face-shields, and N-95 masks). To ensure a proper disinfection, we have evaluated three biological indicators namely *Escherichia coli*, *Mycobacterium smegmatis* and spores of *Bacillus stearothermophilus*, considered as the gold standard for disinfection processes. We next evaluated the impact of repeated VHP treatment on physical features, permeability, and fabric integrity of coveralls and N-95 masks. Next, we performed Scanning Electron Microscopy (SEM) to evaluate microscopic changes in fiber thickness of N-95 masks, melt blown layer or coverall body suits. Considering the fact that any disinfection procedure should be able to meet local requirements, our study included various regionally procured N-95 masks and coveralls available at our institute All India Institute of Medical Sciences (AIIMS), New Delhi, India. Lastly, the practical utility of VHP method developed herein was ascertained by operationalizing a dedicated research facility disinfecting used PPE during COVID-19.

**Results:**

Our prototype studies show that a single VHP cycle (7–8% Hydrogen peroxide) could disinfect PPE and PPE housing room of about 1200 cubic feet (length10 ft × breadth 10 ft × height 12 ft) in less than 10 min, as noted by a complete loss of *B. stearothermophilus* spore revival. The results are consistent and reproducible as tested in over 10 cycles in our settings. Further, repeated VHP treatment did not result in any physical tear, deformity or other appreciable change in the coverall and N-95 masks. Our permeation tests evaluating droplet penetration did not reveal any change in permeability post-VHP treatments. Also, SEM analysis indeed revealed no significant change in fiber thickness or damage to fibers of coveralls or melt blown layer of N-95 masks essential for filtration. There was no change in user comfort and experience following VHP treatment of PPE. Based on results of these studies, and parameters developed and optimized, an institutional research facility to disinfect COVID-19 PPE is successfully established and operationalized with more than 80% recovery rate for used PPE post-disinfection.

**Conclusions:**

Our study, therefore, successfully establishes the utility of VHP to effectively disinfect PPE for a possible reuse as per the requirements. VHP treatment did not damage coveralls, cause physical deformity and also did not alter fabric architecture of melt blown layer. We observed that disinfection process was successful consistently and therefore believe that the VHP-based decontamination model will have a universal applicability and utility. This process can be easily and economically scaled up and can be instrumental in easing global PPE shortages in any biosafety facility or in health care settings during pandemic situation such as COVID-19.

## Introduction

COVID-19 pandemic has impacted global health across the geographies and GDP. The scale, disease aetiology, wide representations ranging from lung to gut, and prolonged incubation period of SARS-CoV-2 virus has created a massive surge in the requirement of personal protective equipment (PPE), especially in health care settings. The disrupted supply lines, and quarantine and lock-down measures implemented across the countries have resulted in acute shortage of quality PPEs, even in the developed and rich nations. Hence, proactive capacity building measures will be urgently required to safely decontaminate the PPEs to enable their selective reuse during contingency situations such as COVID-19 pandemic.

Corona viruses are RNA viruses which have shown susceptibility to a broad range of chemical disinfectants. The chemical disinfectants namely ethanol (78–95%), 2-propanol (70–100%), a combination of 45% 2-propanol with 30% 1-propanol, glutaraldehyde (0.5–2.5%), formaldehyde (0.7–1%) and povidone iodine (0.23–7.5%) readily reduced coronavirus infectivity by approximately 4log_10_ or more [1–4]. Likewise, sodium hypochlorite at a concentration of at least 0.21% and liquid hydrogen peroxide (0.5%, within the incubation time of 1 min) were found to be effective and could deactivate the Severe Acute Respiratory Syndrome (SARS) coronavirus, endemic human coronaviruses (HCoV) or Middle East Respiratory Syndrome (MERS) coronavirus [1–4]. Hydrogen peroxide is an oxidizing agent that works by producing highly reactive hydroxyl radicals that attack nucleic acids and proteins causing viral disintegration. The vaporised form of hydrogen peroxide is much more effective at denaturing proteins as compared to the liquid form [5]. Further, oxidation of DNA/RNA leading to its damage are the mechanisms by which vaporised hydrogen peroxide (VHP) primarily imparts its cidal effect [6]. Moreover, the liquid disinfectant immersion techniques or the wipe-down techniques are often time-consuming, may impact the integrity of the PPE material and thus simply may not be ideal [7].

Gas-based decontamination methods are, therefore, advantageous because of their ability to cover large surface areas and ease of diffusion to difficult to reach areas [8]. Commercial vapor phase hydrogen peroxide (H_2_O_2_) treatments have been developed and investigated for their efficacy in several applications, including decontamination of laboratory and medical equipment, hospital wards and pharmaceutical manufacturing facilities [9]. These methods have been shown to be efficacious against a wide range of organisms, including those producing endospores [10, 11], gram-positive and gram-negative vegetative cells [12, 13], DNA and RNA viruses [14], fungi [13] *Mycobacterium tuberculosis* [15], and phages [16, 17]. Since H_2_O_2_ gets easily decomposed into water and O_2_, the peroxide solution is usually stabilized using various additives such as phosphoric acid, tartaric acid or silver nitrate [18, 19]. Importantly, metal ions such as Ag^+^ not only stabilize highly labile H_2_O_2_ but also have been shown to synergize the antimicrobial effect of H_2_O_2_ [20, 21]. Indeed, several studies have demonstrated the utility of hydrogen peroxide vapors in disinfection of masks and face respirators [17, 22–25]. Nonetheless for PPE disinfection and reuse during pandemics, the method should be robust, have built-in controls for quality assurance and also be optimized to ensure compatibility with a range of PPE (bodysuits, masks and face-shields etc.)

In this study, we evaluate various biological indicators such as the laboratory strains of *Escherichia coli (E. coli)*, *Mycobacterium smegmatis (M. smegmatis),* and spores of *Bacillus stearothermophilus (B. stearothermophilus)* as positive sterilization controls and develop a VHP-based method which successfully decontaminates coveralls, N-95 masks and face shields. We also compare the efficacy of VHP sterilization method with the heat and alcohol-based sterilization. Our experiments clearly establish VHP sterilization as superior to the traditional sterilization methods and hence, can be used for decontaminating PPEs in case of an outbreak such as COVID-19. Further, we have performed droplet permeations tests, and microscopic analysis using Scanning Electron Microscopy (SEM) to show that VHP treatment does not compromise the integrity of the PPE including coveralls and N-95 masks. In summary, our study highlights the utility of VHP-based strategy to ensure a safe and effective disinfection of PPEs for selective reuse. Lastly, using this method, we have successfully established and operationalize a research facility to disinfect PPE used in managing COVID-19 at our institute.

## Materials and methods

### Materials and equipment

Stock Hydrogen peroxide solution (11–12%) stabilized with silver nitrate (0.01%), laboratory grade ethanol, propanol, distilled water, vaporized hydrogen peroxide generator (SATEJ Plus machine, Ahmedabad, India). PPE evaluated in the study include coveralls, N-95 masks, and face-shields obtained for BSL-3 laboratory work, and from hospital supplies of PPE for health workers. We evaluated various concentrations of hydrogen peroxide in this study by diluting the hydrogen peroxide stock to 6, 8 and 10% with distilled water.

### Biological indicators and culture conditions

Biological indicator strips coated with 10^6^ spores of *B. stearothermophilus* spores (Sigma Aldrich, USA) were used as gold standard to affirm the integrity of sterilization process. In addition, saprophytic, non-virulent, recombinant laboratory strains of *E. coli* and *M. smegmatis* (harboring hygromycin resistance marker for selection) were incorporated in the study to evaluate their suitability as biological indicator. Briefly, *E. coli* and *M. smegmatis* were aerobically grown in hygromycin-containing Luria–Bertani (LB) broth, and Middlebrook 7H9 broth (supplemented with 0.05% tween-80 and 10% albumin-dextrose complex), respectively at 37 °C/180 rpm. The treated cultures of *E. coli* and *M. smegmatis* were plated on LB and Middlebrook 7H11 agar (supplemented with 10% albumin-dextrose complex) containing 200 µg/ml and 50 µg/ml hygromycin, respectively. *B. stearothermophilus* spores were grown in Brain Heart Infusion (BHI) media (HiMedia Laboratories, India) at 55 °C/180 rpm.

### Heat and alcohol treatment of bacteria

Aerobically grown *E. coli* and *M. smegmatis* cultures at a cell density of 10^7^ CFU/ml were subjected to different treatments, namely temperature (70 °C and 80 °C for 5 and 10 min each), ethanol (75% and 85% for 0.5 and 1 min each) and propan-2-ol (75% and 85% for 0.5 and 1 min each). The treated cultures were plated on the respective media as described above. The plates were incubated at 37 °C overnight for *E. coli* and 72 h for *M. smegmatis* colonies to appear [26, 27]. The colonies were counted and CFU/ml was compared to untreated culture controls that were also plated in parallel and incubated similarly, and *B. stearothermophilus* spore strips were exposed to 90 °C temperature/30 min, 85% ethanol/1 min or 85% isopropanol/1 min. These were subsequently inoculated in BHI broth and incubated at 55 °C/180 rpm for 72 h. Untreated spores were used as the positive control for these experiments.

### Spiking of the PPE with biological indicators

The areas having high likelihood of contamination during clinical examination were selected to evaluate the efficacy of VHP exposure (collars, chest, arm cuffs and abdomen). The coveralls were hung on copper clothes-line by clips placed near the collar such that there is a space of at least 1 ft between each coverall. These coveralls were spiked at specified areas by inoculation with ~ 10^7^ CFU/ml of *E. coli* and *M. smegmatis*, individually. 100 µl of each bacterial cell suspension were spread uniformly and air dried. *B. stearothermophilus spore* strips were strategically placed at far corners of the room, difficult to access areas, and on each coverall as sterilization indicator. Similarly, the spiking was performed on N-95 masks and face shields, as well as on random surfaces in the room.

### Disinfection procedure/protocol

The room having VHP machine should be clean and dust-free and should have at least 2 doors to ensure separate entry and exit procedures in accordance with the best biosafety practices. We first applied the room parameters (length × breadth × height) into the interface of the VHP generator. The machine allows an automatic calculation of approximate cycle time based on the room volume. For our prototype studies, we used a room size of approximately 10 ft × 10 ft × 12 ft (l × b × h), with the machine run-time of ~ 10 min. The hydrogen peroxide solution was freshly prepared after diluting stock solution and was kept in the designated container in the machine as per the manufacturer’s instructions. Usually for a room size of 1000 cubic feet, approximately 200 ml of final peroxide solution would be needed. Post-VHP cycle, we allowed a retention time of a minimum of 2 h prior to opening the room for collecting the treated PPE. This resting time not only ensures sufficient contact time for peroxide vapors to effectively kill the biological agents but also allows the vapors to diffuse out slowly as they may cause mild user discomfort (skin and eye irritation) if entering the treatment room soon after the runtime is over.

### Validation of disinfection process

To validate the success of disinfection process, we removed the VHP exposed spore strips from bodysuits and all random areas, and inoculated in BHI broth along with control strips (without VHP exposure). Bacteria patched on coveralls and other PPE prior to VHP treatment were retrieved using cotton swabs and thereafter, plated as described. Swabs were also taken post-VHP cycle from random surfaces in the room and inoculated in the relevant medium to confirm room sterilization. The CFU/ml enumerated from the initial unexposed inoculum (time = 0 min) were used as controls.

### Permeation tests of coveralls post-VHP disinfection

The effect of VHP on the integrity of the coveralls was assessed by a liquid permeation/evaporation test periodically after 2nd, 4th, 6th and 8th or higher VHP cycle. We have used water drops of 50 and 100 µl, randomly spotted on the coveralls on high exposure area and calculated the time of permeation through the fiber. To account for environmental evaporation, we spotted an equal amount on a non-absorbent plastic petri dish surface and scored for the time of evaporation. The time taken for water droplet disappearance from PPE surfaces was normalized against droplet evaporation time from the petri dish (i.e. natural evaporation time).

### Scanning electron microscopy

Unexposed and VHP exposed samples (coveralls, N-95 outer layer, N-95 melt blown layer) were gently trimmed (2 × 5 mm) on a clean surface using a scalpel and fixed in 2.5% glutaraldehyde in 0.1 M sodium phosphate buffer (PB, pH 7.4) for 4–6 h at 4 °C. After washing with 0.1 M PB, samples were dehydrated in increasing concentrations of ethanol (30, 50, 70, 80, 90 and absolute) followed by air-drying at room temperature. The samples were mounted on the aluminum stubs such that the outer surface of coverall/mask layer faces up. Thereafter, the samples are coated with gold using the Sputter coating unit, Baltec, Switzerland. Further, the samples were analyzed in a double-blind fashion at the SEM facility AIIMS, New Delhi by using a Zeiss EVO18 SEM microscope, USA, at 200X and 1000X magnification, and morphological features and the width of fibers were noted for analysis.

## Results

### *Determination of the suitability of biological indicators for a PPE disinfection facility for SARS*-*CoV*-*2*

Considering the highly contagious nature of SARS-CoV-2 and reported biosafety considerations/legal guidelines associated with growing a pathogen/viral culture, it is critical to have an appropriate biological indicator that could be used on ground and can act as a surrogate to ensure the optimization of conditions that would be required to operationalize any disinfection facility.

**Fig. 1:**
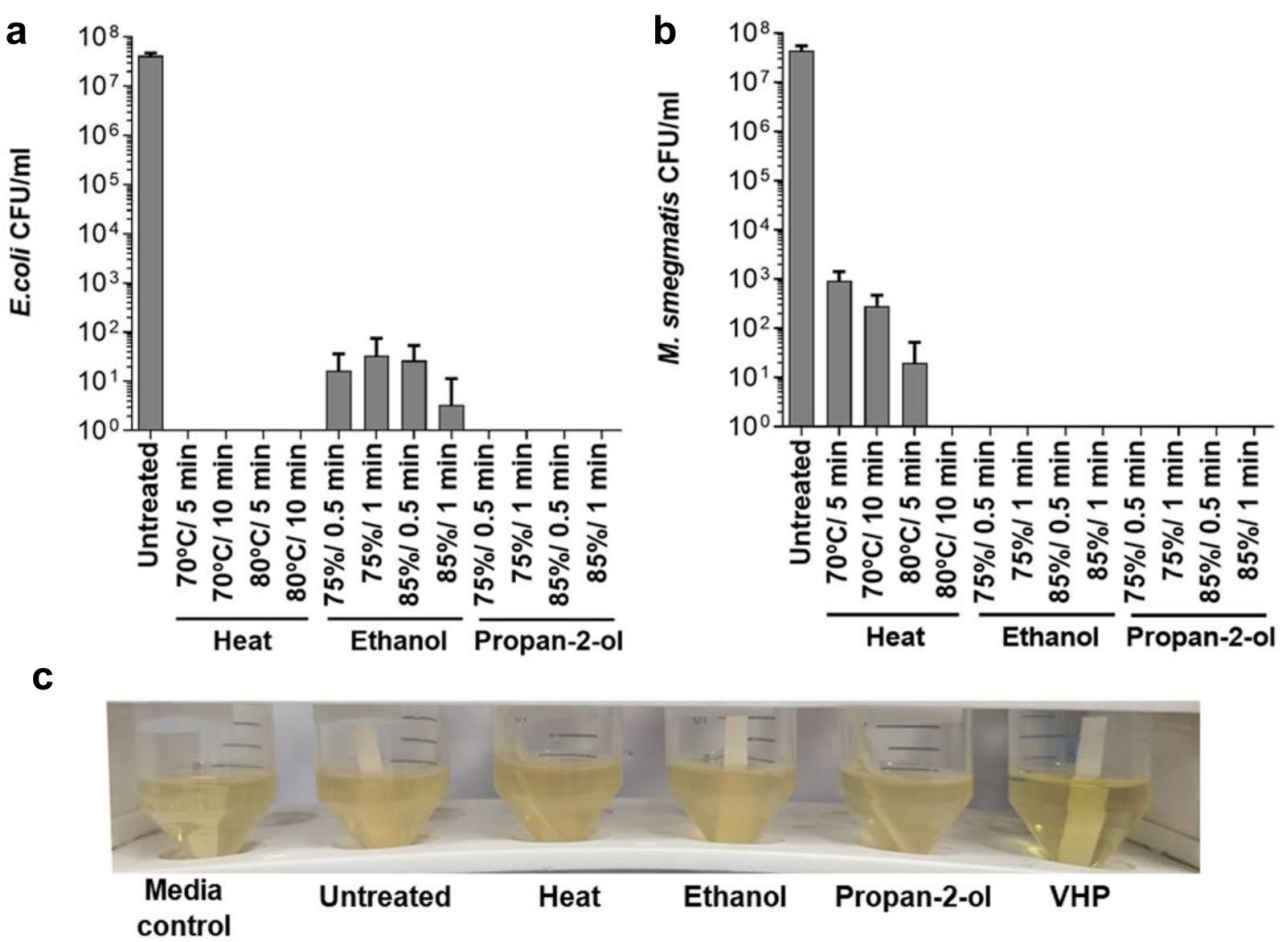
Comparison of heat- and alcohol- based disinfection. **a***E. coli* and **b***M. smegmatis* bacterial cultures were treated with heat (70 °C and 80 °C for 5 and 10 min), ethanol (75% and 85% for 0.5 and 1 min) and propan-2-ol (75% and 85% for 0.5 and 1 min), followed by CFU plating on respective agar media. Untreated culture controls were plated in parallel [****p < 0.0001 obtained from one-way ANOVA with Bonferroni post-test correction]. **c** Representative image of *B. stearothermophilus* spore strips exposed to heat (90 °C/30 min), 85% ethanol (1 min) and 85% propan-2-ol (1 min) inoculated in BHI media. Each experiment was performed at least two times with three biological replicates

Based on the knowledge in literature about virucidal effect of various treatments on SARS-CoV-2, we used the similar parameters to investigate the resilience of candidate biological indicators. In case of *E. coli*, we observed a complete loss of viability at both the temperatures (70 °C and 85 °C) while *M. smegmatis* exhibited some resistance to heat (Fig. 1a, b). Likewise, in the alcohol-based tests, *E. coli* exhibited low level resistance to ethanol, while *M. smegmatis* was non-viable (Fig. 1a, b). Propan-2-ol treatment on the other hand, resulted in loss of viability of both *E. coli* and *M. smegmatis* (Fig. 1a, b). Importantly, under all treatment conditions, we observed at least 6log_10_ reduction in bacterial survival. Nonetheless, the gold standard *B. stearothermophilus* spores exposed to harsh treatments (heat 90 °C/30 min or alcohol 85%/1 min) showed revival and grew well in defined culture conditions (Fig. 1c). It is clear from the data that while candidate biological indicators *E. coli* and *M smegmatis* could show resilience only in a couple of stress tests reportedly used to inactivate SARS-CoV-2, only *B. stearothermophilus* consistently thrived in all the conditions known to inactivate the SARS-CoV-2 virus indicating its versatility as an ideal surrogate or biological indicator to develop disinfection protocols for COVID-19.

### VHP-based disinfection of *E. coli*, *M. smegmatis* and *B. stearothermophilus* spores

**Fig. 2:**
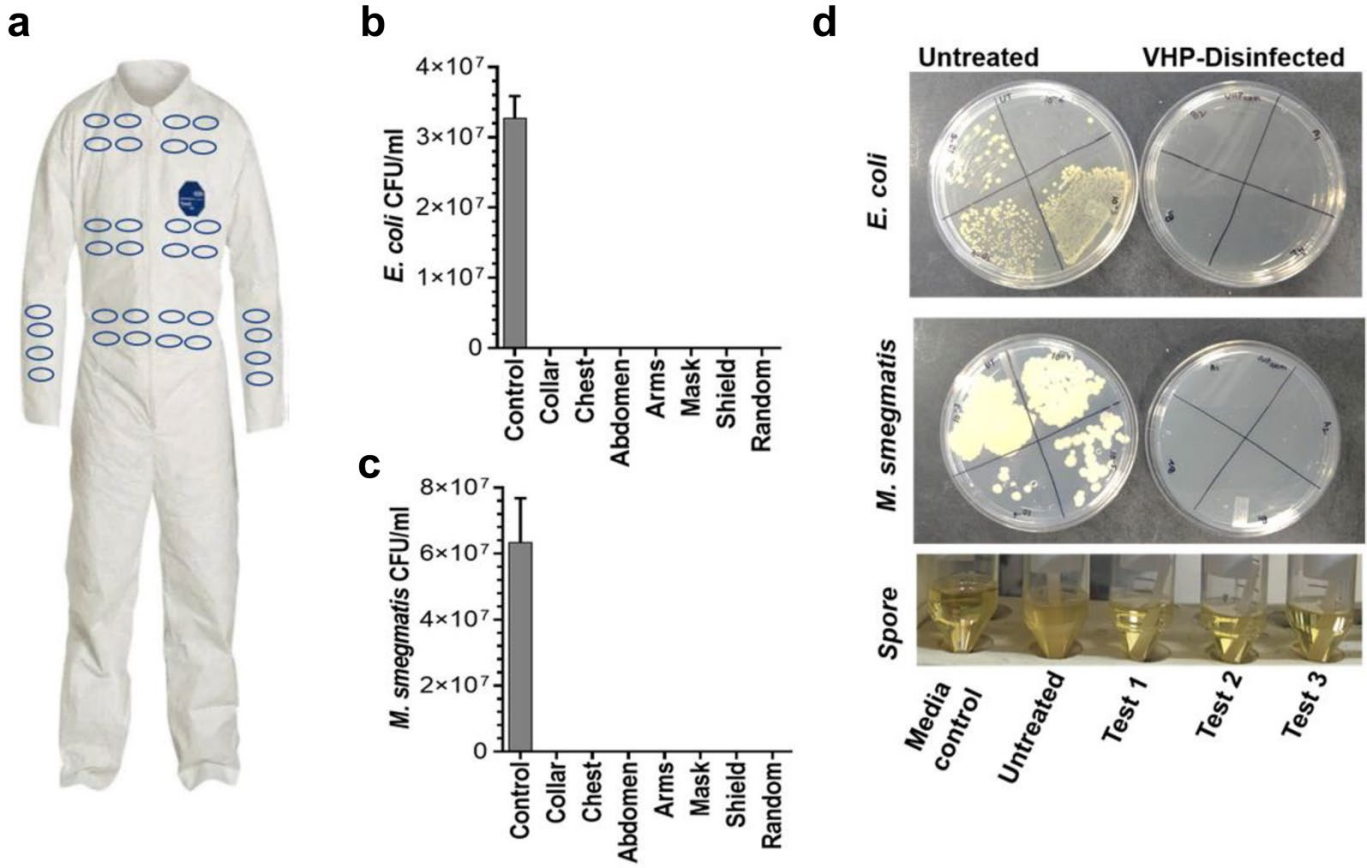
Effect of vaporized hydrogen peroxide on survival of biological indicators. **a** Pictorial depiction of the areas on the coverall inoculated with 10^7^*E. coli* or 10^7^ *M. smegmatis* or *B. stearothermophilus* spores. Blue circles indicate area of high propensity of exposure during clinical examination. After a cycle of VHP-based disinfection, bacteria were retrieved from the coveralls. CFU/ml are shown for the untreated and treated sets of **b***E. coli* and **c***M. smegmatis* plated on respective media. Swabs were taken from random surfaces in the room and plated. Data is represented as an average of at least 8 to 24 biological replicates. **d** Representative image of *E. coli, M. smegmatis* and *B. stearothermophilus* spores untreated vs VHP exposed, plated and inoculated on respective growth media. (N = 10). [****p < 0.0001 obtained from one-way ANOVA with Bonferroni post-test correction]

We next evaluated how the three candidate biological indicators perform *vis a vis* VHP disinfection treatment. For this, we spiked the body-suits/coveralls, face-shields, masks and random surfaces with different biological indicators based on the likelihood of their exposure during clinical examination (Fig. 2a). Following VHP exposure, we observed a complete sterilization of *E. coli* and greater than 7 log_10_ reduction in *M. smegmatis* CFU post-exposure to VHP (Average colony count < 1, across various runs) (Fig. 2b–d). Most importantly, *B. stearothermophilus* spores exposed to VHP failed to revive in BHI media indicating the success of the disinfection process (Fig. 2d).

### *Permeability tests and scanning electron microscopy to determine the integrity of coveralls and N*-*95 masks*

**Fig. 3:**
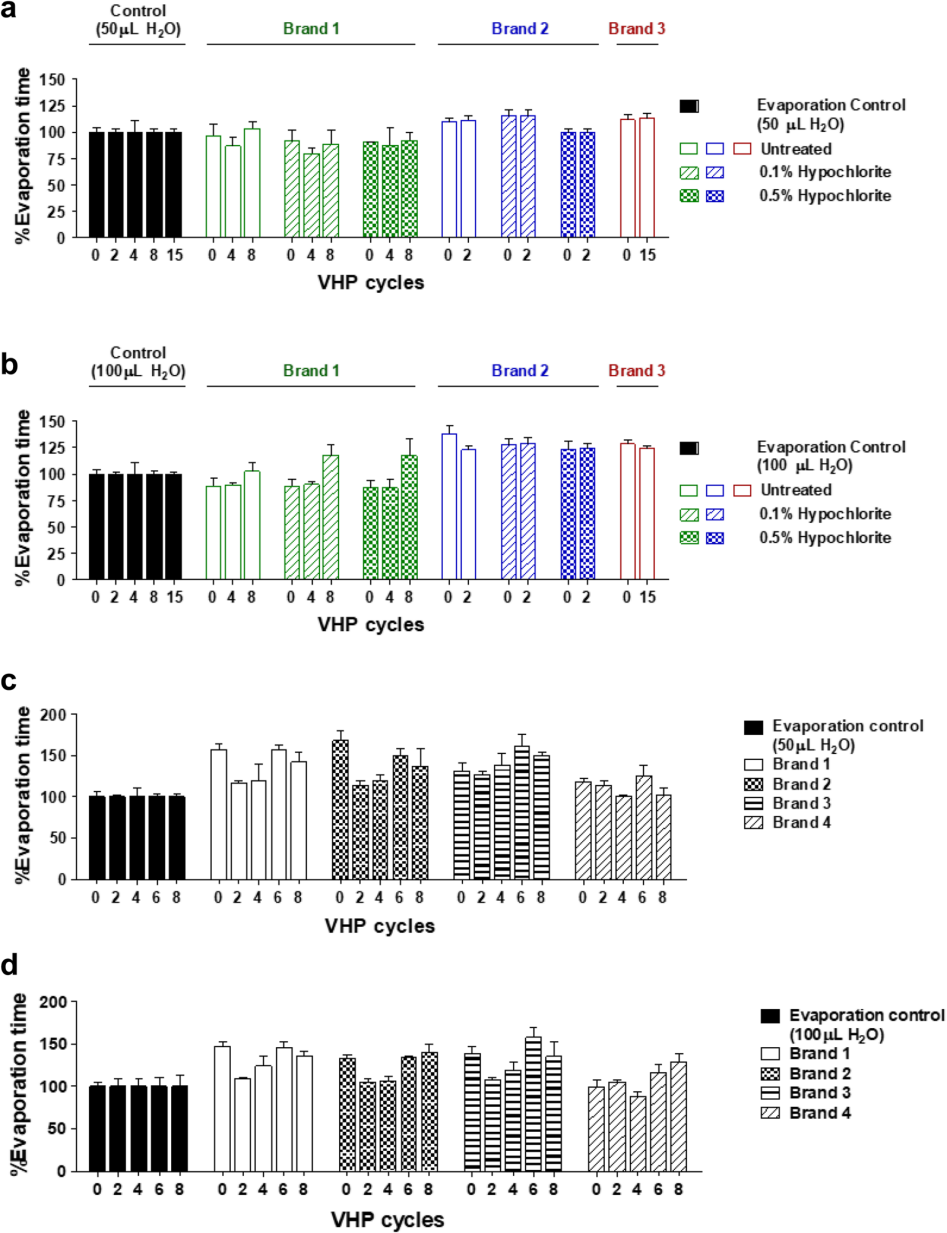
Analysis of integrity of selected brands of coveralls and N-95 masks following multiple cycles of VHP exposure by liquid permeation test. Using water droplets of different volume, we investigated the change in permeability status of coveralls arising due to multiple cycles of VHP exposure. **a**, **b** Coveralls**. c, d** N-95 masks. 50 µL (**a**, **c**) and 100 µL (**b**, **d**) water was seeded on selected brands of coveralls and N-95 masks. Hard non-permeable surface (petri plate surface) was used as control for evaporation. The time taken for water droplet disappearance from PPE surfaces was normalized against droplet evaporation time from the petri dish (i.e. natural evaporation time). Time comparable to or slightly higher to evaporation control in tested coveralls and N-95 masks indicate no permeation associated loss of droplet indicating integrity of PPE. Lack of availability of some suits limited a full range of analysis on some datasets. [Statistical evaluation done by Two-way ANOVA, with Bonferroni post-test correction]. Clearly, VHP treatment did not result into significant changes in permeability of PPE

After establishing the disinfection potential of VHP, we next determined whether VHP treatment impacts integrity of coveralls, masks or other PPE items. First of all, we did not observe any physical tear or deformity in the coveralls or N-95 masks or any blurriness/opaqueness in the face-shields to indicate any deformity. Further, we did not observe any significant change in the permeability of droplets on various coveralls and N-95 mask layers post-VHP treatment (Fig. 3a–d). The integrity of the coveralls or masks did not show any discernable alteration following hypochlorite treatment, which may be essential sometimes to remove stains of body fluids post-VHP disinfection.

**Fig. 4:**
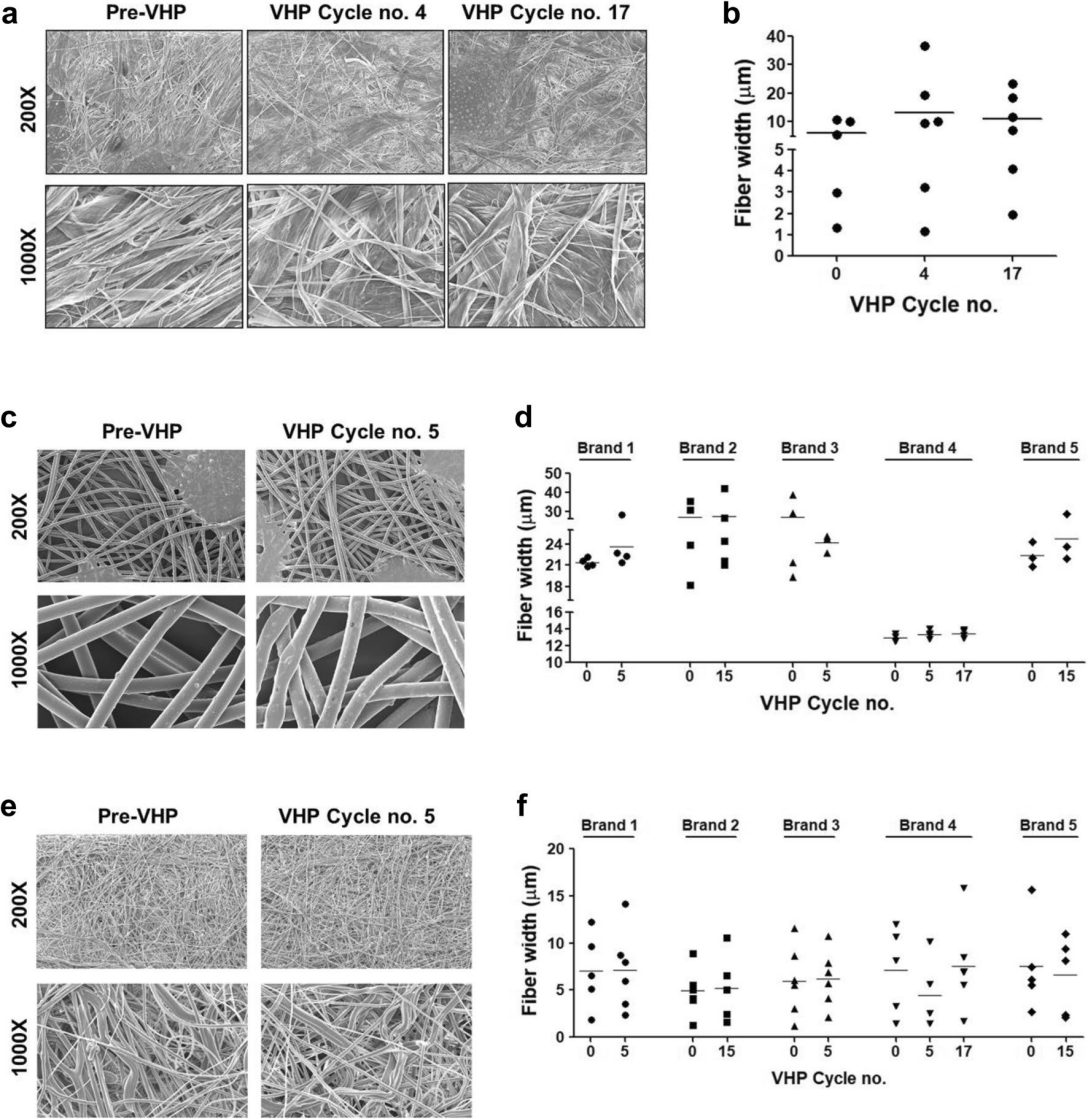
Analysis of integrity of selected brands of coveralls and N-95 masks following multiple cycles of VHP exposure by scanning electron microscopy. **a** Representative SEM photomicrograph of a brand of coverall at 200X and 1000X magnification pre- and post-multiple cycles of VHP exposure. **b** Comparison of fibre width of selected brand of coverall, before and after VHP exposure. **c** Representative SEM photomicrograph of a brand of N-95 mask outer layer at 200X and 1000X magnification pre- and post-5 cycles of VHP exposure. **d** Comparison of fiber width of the outer layer of selected brands of N-95 masks, before and after VHP exposure. **e** Representative SEM photomicrograph of a N-95 mask’s melt blown layer at 200X and 1000X magnification, pre- and post- 5 cycles of VHP exposure. **f** Comparison of fiber width of the melt blown layer of selected brands of N-95 masks, pre- and post- VHP exposure [Statistical evaluation done by two-way ANOVA, with Bonferroni post-test]. No significant differences observed due to VHP treatment either in external layer of mask or in the melt blown layer of the N-95 mask. Similarly, there were no discernable changes observed in integrity of coveralls

Next to evaluate whether VHP treatment impacts microscopic integrity of coveralls or N-95 masks, we performed a SEM analysis investigating fiber texture/morphological changes and fiber thickness. This analysis is especially relevant for N-95 masks, in which melt blown layer is essential for proper filtration. As shown in Fig. 4, VHP treatment did not cause any significant alterations to the microscopic structure of coveralls (Fig. 4a, b), N-95 masks outer layer (Fig. 4c, d) or melt blown layer (Fig. 4e, f) critical to ensure respiratory protection. Even repeated cycles (n > 15) of VHP treatment did not impact the integrity of masks or coveralls justifying the suitability of VHP as a preferred method for disinfection.

### Operationalizing the PPE disinfection research facility

Using the parameters and quality control developed and optimized in this study, a VHP-based PPE disinfection research facility (~ 1200 sq ft) for selective reuse is recently commissioned at the institute with dedicated areas including independent donning and doffing zones. Since the commissioning (~ 4 weeks), we have successfully accomplished the disinfection of over 2000 PPE coveralls, and similar number of N-95 masks used in COVID-19 ICU and other hospital areas. Our rate of recovery of functional PPE post-VHP disinfection ranges from 80 to 85%, thereby rationalizing the establishment of such facilities in health care settings. Hence, such onsite facilities could be instrumental in ensuring a regular supply of PPE for health care workers during the pandemic associated times of lockdown or disruption of supply lines.

## Discussion

The need to have a safe, effective and simple protocol adaptable to the local conditions and materials cannot be overstated in the time of global pandemic such as COVID-19. The challenge for developing and mid-income countries is especially acute as global shortage has skyrocketed the prices, thereby limiting the reach of PPE to the countries which probably need them on urgent basis. It is prudent that countries have locally adapted and customized protocols in hand to disinfect the PPEs for a judicious reuse during contingency measures.

Firstly, we show that VHP treatment completely cleared two candidate biological indicators namely *E. coli* and *M. smegmatis.* The idea to evaluate *E. coli* or *M. smegmatis* as biological indicators stemmed from the need to accelerate the confirmation of a successful disinfection cycle following a VHP run as these organisms are fairly resilient compared to viruses [28] and have relatively faster growth [*E. coli* (< 1 day) and *M. smegmatis* (~ 3 days)] [26, 27]. In fact, there is an established hierarchy among microbes based on their resilience to chemical disinfection processes [28, 29]. The spores are the most resilient microbial entity to disinfection methods including VHP treatment, followed by mycobacterial strains, but usually require more time to grow (4–5 days). We expect that VHP treatment conditions which are optimized to clear mycobacteria and spores in our studies would also be effective against less resistant classes of microorganisms, such as viruses including SARS-CoV-2. Furthermore, consistent with our goal to establish an operational PPE disinfection unit, we were mindful of the requirement of a biological control whose clearance would indicate success of the disinfection cycle. The culturing of COVID-19 etiological agent SARS-CoV-2 has an operational requirement of a BSL-3. Hence it was prudent to optimize the disinfection parameters using a biological indicator which has a higher degree of resilience to chemical disinfection than SARS-CoV-2, while being amenable to routine application as indicator in an operational PPE Disinfection Unit without stringent biosafety requirements.

Based on the above premise, we sought to develop and optimize conditions that are sufficient to deactivate the biological indicators of the highest resilience. The two bacterial strains showed only moderate resilience at parameters known to kill SARS-CoV-2, while spores grew without any deficit in these conditions (Fig. 1). This data therefore highlighted the relevance of spores, and indicated of limited utility of two bacterial strains to act as a reliable indicator of PPE disinfection in COVID-19-facility. Hence, we optimized the conditions that consistently demonstrated a 100% deactivation of *B. stearothermophilus* spores spiked at high exposure area on coveralls and N-95 masks (Fig. 2) using a final hydrogen peroxide concentration of 6%, 8% and 10% prepared by diluting the original stock solution/reagent with distilled water. The three concentrations of H_2_O_2_ tested worked with the following observations: A 6% Hydrogen peroxide is the lower threshold that will produce desirable disinfection. However, any mishandling, leak or degradation of peroxide solution due to poor storage has the potential to compromise the effective dosage and hence the process. At 10% hydrogen peroxide, we observed that machine requires extensive cleaning after every cycle, often not feasible in the operational settings; or else the vaporization nozzle of the machine may get fully or partially clogged yielding unsatisfactory results. In our experience, 8% hydrogen peroxide solution provides the perfect balance in consistently ensuring complete disinfection without significantly increasing the cost.

Next, to determine whether our disinfection approach causes any physical deformity, or weakness of fiber strength that could compromise PPE function, we performed macroscopic and microscopic analysis on the VHP treated PPE. First, we did not observe any physical tear or deformity in the coverall, N-95 masks or face-shields. A double blinded experiment wherein volunteers from laboratory staff wore both new and VHP treated PPE did not highlight any significant differences in the user experience with respect to fit or comfort (data not shown). Cognizant of the fact that the user experiences may be prone to subjective biases, we performed in-house permeation tests and SEM studies which really substantiated that VHP-based disinfection process did not result into macroscopic and microscopic alterations. Especially, SEM analysis did not reveal any change in fiber thickness or any other apparent damage to fibers or to melt blown layers even after repeated VHP treatment. For a couple of the VHP treated suits subjected to a synthetic blood penetration test, the results were consistent with the findings of in-house permeations tests and SEM analysis and did not reveal any significant change in the blood permeability of bodysuits even after more than 15 cycles of VHP treatment (data not shown). Therefore, our study significantly advances the PPE disinfection and reuse field including the operationalization of the established protocols in COVID-19 clinical settings. Previous studies on VHP have primarily focused on efficiency of disinfection of respiratory protection, i.e. masks and face respirators [17, 22–25]. Our study, on the other hand, have focused on the effect of VHP treatment on a range of PPE including the full body suits/coveralls, face shields, and various layers of N-95 masks including the inner melt blown layer to study fiber integrity and texture using SEM. The permeation studies and synthetic blood barrier test has highlighted that repeat VHP treatment does not alter the integrity of body suits and masks. Our successful disinfection studies and choice of multiple locally available brands ensures a wide spread utility of the VHP-based method. Lastly, the highest resilience of spores and their selection as biological indicator for disinfection abrogates the need of having a pandemic specific microbe for setting-up any new VHP-based disinfection facility for PPE reuse during outbreaks.

The application of VHP method has significant merits over other known methods of disinfection such as UV light. The effectiveness of other disinfection approaches such as UV light varies depending upon viral load, nature of contaminated surface, distance from the source and uniformity of the radiation [24]. Further UV light is known to impact cross linking of fiber [30, 31] and degradation of filter performance in case of N-95 masks, and strength in case of coveralls. Likewise, heat has also been used limitedly but application of heat is known to compromise the filtration capacity [25]. Another widely used method in hospitals utilizes ethylene oxide gas, which is less safe than hydrogen peroxide vaporization, less environment friendly, inflammable and potentially carcinogenic [32, 33]. Moreover, a prolonged period of aeration following item exposure to the gas is required to eliminate chemical residua. This results in an extremely long cycle time of more than 20 h compared to nearly 2 h for VHP [32, 33].

Lastly, to meet the operational requirements in the field, the process has to be effective at a wide temperature range. In our experiments, we have run the VHP treatment at room temperature (no air conditioning) with temperature readings varying from 25 °C to 38 °C across various runs conducted over a month. This variation in temperature did not impact the quality of results of disinfection process. Finally, the disinfection was effective even in the remote inaccessible corners as demonstrated by lack of growth of biological indicators picked from the farthest corners of the room or areas farthest from the machine. Use of a VHP disinfection machine can be scaled to permit simultaneous sterilization of a large number of used PPE. A prototype room of ~ 1200 cubic feet (10 ft × breadth 10  ft × height 12 ft) could process over 1000 N-95 masks or ~ 25–30 coveralls in less than 10 min of operational time of VHP machine. Taken together, our data show that VHP sterilization allows the best combination of rapid decontamination/disinfection and preservation of coverall and masks’ integrity.

From an operational aspect, we believe that the following key steps must be taken to ensure a proper functioning and usage of disinfected PPE:

1. It must be ensured that PPE heavily soiled with patient fluids or physically damaged (torn coverall or masks with broken elastics) are to be discarded at the doffing site itself as per the Institutional guidelines.
2. Integrity of the disinfection process must be ensured with appropriate quality control for individual treatment cycle. We recommend using *B. stearothermophilus* spores as biological indicator for this purpose.
3. Following a successful disinfection, the PPE must be checked for its integrity, fit and strength.
4. User experience should be factored in determining the suitability of PPE in question for potential reuse.

Following these guidelines, we have successfully processed more than 2000 PPE bodysuits used in COVID-19 hospital areas with a post-disinfection recovery rate for functional PPE to be > 80%. This high recovery rate using VHP disinfection is indicative of environmental and economical sustainability. A rough offhand calculation revealed the cost of PPE disinfection in our facility to be less than a quarter dollar/coverall. A large facility will allow more suits and masks to be disinfected and will further reduce the cost. Lastly, the VHP machine can also be used to sterilize BSL-3 facilities, tissue culture rooms or other laboratories or hospital areas and thus would have a great utility to discount the one-time cost of machine (~ 4000$).

## Conclusion

Any intended method developed to enable a selective reuse of PPE should be able to meet a couple of broad requirements. Not only it should be able to disinfect the used PPE but also it should not negatively impact the integrity and functionality of treated PPE. In addition, the method should have the potential to be easily and economically scaled up. Our results show that VHP-based disinfection method successfully fulfils these requirements and therefore is a suitable process to ensure a safe and effective reuse of PPEs. Considering the compatibility of VHP-based disinfection method to a broad range of PPE make and types (Masks, coveralls, face-shields), we believe that VHP-based decontamination protocol will have a universal applicability and utility in mitigating the shortages of PPE in situations like that of COVID-19.

## Data Availability

Not applicable.

## References

[1] Kampf G, Todt D, Pfaender S, Steinmann E (2020). Persistence of coronaviruses on inanimate surfaces and their inactivation with biocidal agents. J Hosp Infect.

[2] Rabenau HF, Kampf G, Cinatl J, Doerr HW (2005). Efficacy of various disinfectants against SARS coronavirus. J Hosp Infect.

[3] Rabenau HF, Cinatl J, Morgenstern B, Bauer G, Preiser W, Doerr HW (2005). Stability and inactivation of SARS coronavirus. Med Microbiol Immunol.

[4] Siddharta A, Pfaender S, Vielle NJ, Dijkman R, Friesland M, Becker B, Yang J, Engelmann M, Todt D, Windisch MP, Brill FH (2017). Virucidal Activity of World Health Organization-Recommended Formulations Against Enveloped Viruses, Including Zika, Ebola, and Emerging Coronaviruses. J Infect Dis..

[5] Finnegan M, Linley E, Denyer SP, McDonnell G, Simons C, Maillard JY (2010). Mode of action of hydrogen peroxide and other oxidizing agents: differences between liquid and gas forms. J Antimicrob Chemother.

[6] Linley E, Denyer SP, McDonnell G, Simons C, Maillard JY (2012). Use of hydrogen peroxide as a biocide: new consideration of its mechanisms of biocidal action. J Antimicrob Chemother.

[7] Krause J, McDonnell G, Riedesel H (2001). Biodecontamination of animal rooms and heat-sensitive equipment with vaporized hydrogen peroxide. J Am Assoc Lab Anim Sci.

[8] Rogers JV, Choi YW, Richter WR, Stone HJ, Taylor ML (2008). Bacillus anthracis spore inactivation by fumigant decontamination. Appl Biosaf..

[9] Goyal SM, Chander Y, Yezli S, Otter JA (2014). Evaluating the virucidal efficacy of hydrogen peroxide vapour. J Hosp Infect.

[10] Kokubo M, Inoue T, Akers J (1998). Resistance of common environmental spores of the genus Bacillus to vapor hydrogen peroxide. PDA J Pharm Sci Technol.

[11] Barbut F, Menuet D, Verachten M, Girou E (2009). Comparison of the efficacy of a hydrogen peroxide dry-mist disinfection system and sodium hypochlorite solution for eradication of Clostridium difficile spores. Infect Control Hosp Epidemiol.

[12] McDonnell G, Russell AD (2001). Antiseptics and disinfectants: activity, action, and resistance. Clin Microbiol Rev.

[13] Meszaros JE, Antloga K, Justi C, Plesnicher C, McDonnell G (2005). Area fumigation with hydrogen peroxide vapor. Appl Biosafety..

[14] Heckert RA, Best M, Jordan LT, Dulac GC, Ed-dington DL, Sterritt WG (1997). Efficacy of va-porized hydrogen peroxide against exotic animal viruses. Appl Environ Microbiol..

[15] Hall L, Otter JA, Chewins J, Wengenack NL (2007). Use of hydrogen peroxide vapor for deactivation of *Mycobacterium tuberculosis* in a biological safety cabinet and a room. J Clin Microbiol..

[16] Mentel R, Shirrmakher R, Kevich A, Dreĭzin RS, Shmidt I (1977). Virus inactivation by hydrogen peroxide. Voprosy virusologi..

[17] Kenney P, Chan BK, Kortright K, Cintron M, Havill N, Russi M, Epright J, Lee L, Balcezak T, Martinello R. Hydrogen Peroxide Vapor sterilization of N95 respirators for reuse. MedRxiv. 2020 . 10.1017/ice.2021.48 PMC8185421 33557979

[18] Martin NL, Bass P, Liss SN (2015). Antibacterial properties and mechanism of activity of a novel silver-stabilized hydrogen peroxide. PLoS ONE.

[19] Kelly F, Mckay C, Steed BH. Solutions for stabilizing hydrogen peroxide containing solutions. GB patent no. PCT/GB1990/001968. 1991.

[20] Pedahzur R, Shuval HI, Ulitzur S (1997). Silver and hydrogen peroxide as potential drinking water disinfectants: their bactericidal effects and possible modes of action. Water Sci Technol.

[21] Davoudi M, Ehrampoush MH, Vakili T, Absalan A, Ebrahimi A (2012). Antibacterial effects of hydrogen peroxide and silver composition on selected pathogenic enterobacteriaceae. Int J Env Health Eng..

[22] Hao L, Wu J, Zhang E, Yi Y, Zhang Z, Zhang J, Qi J (2019). Disinfection efficiency of positive pressure respiratory protective hood using fumigation sterilization cabinet. Biosafety Health..

[23] Viscusi DJ, Bergman MS, Eimer BC, Shaffer RE (2009). Evaluation of five decontamination methods for filtering facepiece respirators. Ann Occup Hyg.

[24] Torres AE, Lyons AB, Narla S, Kohli I, Parks-Miller A, Ozog D, Hamzavi IH, Lim HW. Ultraviolet-C and other methods of decontamination of filtering facepiece N-95 respirators during the COVID-19 pandemic. Photochem Photobiol Sci. 2020. 10.1039/d0pp00131g PMC8047514 33856682

[25] Rubio-Romero JC, del Carmen Pardo-Ferreira M, García JA, Calero-Castro S (2020). Disposable masks: Disinfection and sterilization for reuse, and non-certified manufacturing, in the face of shortages during the COVID-19 pandemic. Saf Sci..

[26] Saini V, Raghuvanshi S, Talwar GP, Ahmed N, Khurana JP, Hasnain SE, Tyagi AK, Tyagi AK (2009). Polyphasic taxonomic analysis establishes *Mycobacterium indicus pranii* as a distinct species. PLoS ONE..

[27] Ahmed N, Saini V, Raghuvanshi S, Khurana JP, Tyagi AK, Tyagi AK, Hasnain SE (2007). Molecular analysis of a leprosy immunotherapeutic bacillus provides insights into Mycobacterium evolution. PLoS ONE..

[28] Rickloff, J, Orelski, P. Resistance of various microorganisms to vapor phase hydrogen peroxide in a prototype dental hand piece/general instrument sterilizer, abstr. Q-59. In Abstr. 89th Annu Meet Am Soc Microbiol. 1989; 339.

[29] U.S. Food & Drug Administration. Enforcement Policy for Sterilizers, Disinfectant Devices, and Air Purifiers during the Coronavirus Disease 2019 (COVID-19) Public Health Emergency. Guidance for Industry and Food and Drug Administration Staff. U.S. Department of Health and Human Services, Food and Drug Administration, Centre for Devices and Radiological Health. 2020.

[30] Card KJ, Crozier D, Dhawan A, Dinh M, Dolson E, Farrokhian N, Gopalakrishnan V, Ho E, Jagdish T, King ES, Krishnan N (2020). UV sterilization of personal protective equipment with idle laboratory biosafety cabinets during the Covid-19 pandemic. MedRxiv..

[31] Lindsley WG, Martin SB, Thewlis RE, Sarkisian K, Nwoko JO, Mead KR, Noti JD (2015). Effects of ultraviolet germicidal irradiation (UVGI) on N-95 respirator filtration performance and structural integrity. J Occup Environ Hyg..

[32] Mendes GCC, Brandão TRS, Silva CLM (2007). Ethylene oxide sterilization of medical devices: a review. Am J Infect..

[33] Jinot J, Fritz JM, Vulimiri SV, Keshava N (2018). Carcinogenicity of ethylene oxide: key findings and scientific issues. Toxicol Mech Method..

